# The "AVA - organ"

**DOI:** 10.1186/2046-7648-4-S1-A95

**Published:** 2015-09-14

**Authors:** Leif Vanggaard, Kalev Kuklane, Amitava Halder

**Affiliations:** 1 Danish Arctic Institute, Copenhagen, Denmark; 2 The Thermal Environment Laboratory, Department of Design Sciences, Lund University, Lund, Sweden

## Introduction

It has been shown that the cutaneous arteriovenous anastomoses (AVAs) in hands and feet play a crucial role in the moment to moment regulation of man's body temperature [1]. That role depends, however, on how much of the total body surface that is available to the influence of the AVAs. As the AVAs drain to the superficial veins of the dorsal hand, up along the forearm until these veins disappear into the depth at the upper arm, the skin area for heat dissipation is around 30-40% of the total skin surface. It is this system we have proposed to call "The AVA-organ".

## Methods

In order to substantiate this we have used IR-photos (Flir T200, Sweden) together with thermistors that give the surface skin temperatures of the hands and forearms.

## Results

**Figure 1:**
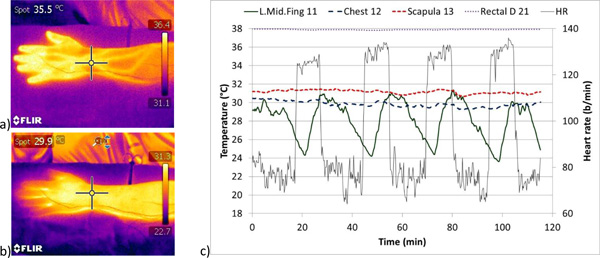
**IR-photos of the arm of a) warm, b) cold person and c) temperature curves under with intermittent exercise**.

In the IR-photos (Figure 1 and 1b) it is seen that the surface skin temperatures are high over the superficial veins of the AVA-organ in the warm person, while in the cold exposed person these veins disappear into the background of the heat that is brought to the surface from the underlying structures. The function of AVAs is demonstrated in an experiment with intermittent exercise in a cool room (Figure 1c).

## Discussion

The distribution of heat shows that in warm man the AVA-organ is the sole part of the surface skin that actively tends to counteract an overheating. This is a new way of seeing the role of the AVAs. This is further demonstrated by the passive temperature decrease in all other skin areas.

## Conclusion

The arteriovenous anastomoses together with the superficial venous retes in the hand, forearm and parts of the upper arm and the similar structures in the leg may be described as an organ, the AVA-organ. As no similar and synchronous surface temperature changes, to those of the proposed organ, are found in other skin areas of the body, the AVA-organ should be regarded as the main moment to moment regulator of the physical heat exchange over the skin.
